# A Machine Learning Pipeline for Gait Analysis in a Semi Free-Living Environment

**DOI:** 10.3390/s23084000

**Published:** 2023-04-14

**Authors:** Sylvain Jung, Nicolas de l’Escalopier, Laurent Oudre, Charles Truong, Eric Dorveaux, Louis Gorintin, Damien Ricard

**Affiliations:** 1 Université Paris Saclay, Université Paris Cité, ENS Paris Saclay, CNRS, SSA, INSERM, Centre Borelli, F-91190 Gif-sur-Yvette, France; 2 Université Sorbonne Paris Nord, L2TI, UR 3043, F-93430 Villetaneuse, France; 3 AbilyCare, 130 Rue de Lourmel, F-75015 Paris, France; 4 ENGIE Lab CRIGEN, F-93249 Stains, France; 5 Université Paris Cité, Université Paris Saclay, ENS Paris Saclay, CNRS, SSA, INSERM, Centre Borelli, F-75006 Paris, France; 6 Service de Neurologie, Service de Santé des Armées, HIA Percy, F-92190 Clamart, France; 7 Novakamp, 10-12 Avenue du Bosquet, F-95560 Baillet en France, France; 8 Ecole du Val-de-Grâce, Service de Santé des Armées, F-75005 Paris, France

**Keywords:** free-living, wearable sensor, IMU, graphical feedback, change point detection, Human Activity Recognition

## Abstract

This paper presents a novel approach to creating a graphical summary of a subject’s activity during a protocol in a Semi Free-Living Environment. Thanks to this new visualization, human behavior, in particular locomotion, can now be condensed into an easy-to-read and user-friendly output. As time series collected while monitoring patients in Semi Free-Living Environments are often long and complex, our contribution relies on an innovative pipeline of signal processing methods and machine learning algorithms. Once learned, the graphical representation is able to sum up all activities present in the data and can quickly be applied to newly acquired time series. In a nutshell, raw data from inertial measurement units are first segmented into homogeneous regimes with an adaptive change-point detection procedure, then each segment is automatically labeled. Then, features are extracted from each regime, and lastly, a score is computed using these features. The final visual summary is constructed from the scores of the activities and their comparisons to healthy models. This graphical output is a detailed, adaptive, and structured visualization that helps better understand the salient events in a complex gait protocol.

## 1. Introduction

Portable inertial sensors such as accelerometers, gyroscopes, or Inertial Measurement Units (IMUs) are frequently used to analyze human activity. In particular, gait quantification is a major subject of interest for clinicians, as it can help to precociously detect the risk of falling or be applied in the context of longitudinal follow-up for degenerative diseases [1]. Most of the published studies are led in clinical and controlled environments (laboratories, etc.), where efficient algorithms have been developed to extract from the raw data relevant features such as gait events, with precision as low as a few milliseconds [2,3,4,5,6].

However, these environments can induce a Hawthorne effect (“white coat” effect) on the patients and therefore bias the gait analysis process [7,8]. Indeed, during controlled studies, subjects are aware of being measured and this implies over- or under-performance during the protocol. A study has shown for instance that the variability of step duration in a free or semi-controlled environment is statistically different from the one measured in a controlled environment [9]. These effects can be due to the bulkiness of the sensors, to the instructions given to the recorded subjects, or to the narrowness of the measurement environments. Moreover, gait cannot be fully apprehended and analyzed without acknowledging phenomena caused by free-living measurements (fatigue, open spaces). A wide range of new protocols, referred to as Free-Living Environments (FLEs) protocols have therefore emerged to avoid these issues and to improve our understanding of human behavior in FLEs, or in the “wild” [10].

The study of locomotion is more challenging in FLEs than in controlled settings, as the automatic computation of gait features at a micro level in FLE (stride times, step times) is more complex when no context information is available [11]. As for the current studies set up by the scientific community in FLEs, they present very diversified goals. The vast majority of studies carried out in FLEs aim to perform Human Activity Recognition (HAR) and to use its outputs to enable clinical analyses [12,13]. HAR can thus help providing general metrics about activity durations in order to enable a quantified follow-up of patients’ physical activity. This can also be the first step in estimating a patient’s energy expenditure over long periods. Indeed, some works aim specifically to measure the time spent in activities that require a greater or lesser expenditure of energy with or without using HAR [14,15,16]. Activities are then associated with a metabolic equivalent of task which is the objective measure of a participant’s rate of energy expenditure in relation to mass. Finally, another large group of studies is dedicated to the detection of falls in FLEs in order to respond to the public health issues that these falls represent [17,18].

In almost all of the work cited above, the output measures provided to clinicians are often generalized/aggregated through simple features. For instance, HAR studies tend to only determine the time spent in various targeted activities, whereas studies that focus on calculating energy expenditure only quantify the time spent in more or less energy-consuming activities. Works that seek to detect falls rely on general output metrics as well (number of falls, time of falls). This, therefore, leads to analyses of physical activity summarized by aggregate and averaged output measures that may hide some relevant phenomena of interest. To get around this drawback, one idea would be to use fine-grained features such as those used in clinical settings. However, this would imply a greater computational burden (e.g., detection of all steps, strides) and a prohibitive flow of information that would overwhelm clinicians and prevent them from obtaining a clear and quickly understandable assessment of the physical activity of their patients. Based on this observation, we present in this paper an alternative and intermediate solution whose major innovation is to provide an accurate macro-analysis with a low computational cost and which is ergonomic for clinicians. This visual summary also allows to keep a temporal structure that helps to provide new interpretations to the free-living recordings (impact of fatigue on specific regimes, performance during transitions between long and short walking regimes).

To the authors’ knowledge, few, if any studies based on the use of IMUs in FLEs have endeavored to provide a macro-analysis displayed in the form of an easy-to-understand visual summary that fully assesses the entire timeline of FLE signals. The purpose of our paper is to describe the processing steps to compute such accurate and didactic quantitative feedback on a subject’s walking during an acquisition in a Semi Free-Living Environment (Semi FLE). We believe that such visualization will help clinicians to perform more accurate and comprehensive longitudinal tracking of locomotion in the natural environment of their patients. This could for instance allow us to evaluate rehabilitation procedures or treatment choices for specific diseases and to assess the impact of treatments on pathologies such as musculoskeletal tumors of the lower limbs or neurological disorders (Parkinson’s disease for instance). The current monitoring of the effects of these FLE treatments is only carried out via calculations of general metrics (steps/day, ambulatory bouts/day) [19] when our graphical tool will enable a refined follow-up displaying an enhanced macro analysis of gait phases.

This paper is organized as follows. Section 2 describes the protocols and algorithms used to create the graphical tool in question. Section 3 provides a comprehensive evaluation of each of the steps of the processing pipeline as well as some results obtained on both pathological and healthy subjects. In Section 4, these results are analyzed and discussed.

## 2. Material and Methods

**Figure 1:**
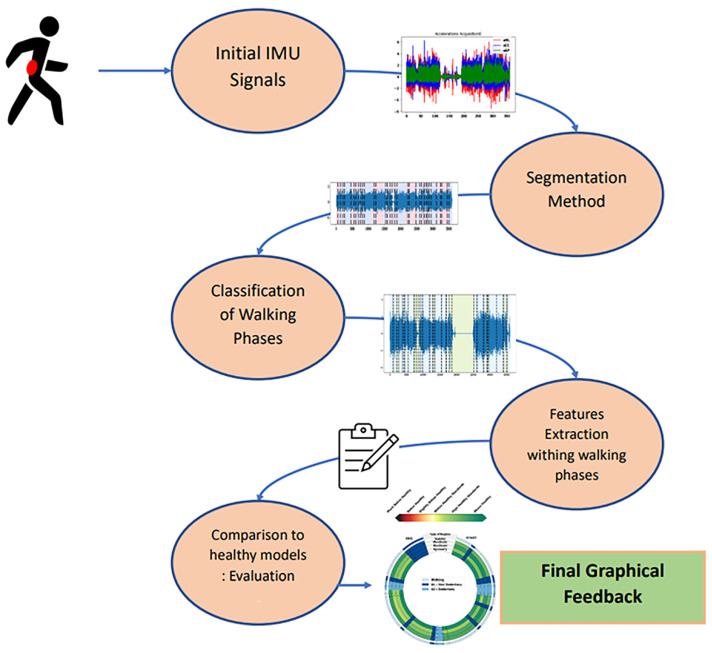
Successive steps of our processing pipeline to render a graphical feedback from a semi-FLE acquisition.

The proposed pipeline is composed of four successive steps: data segmentation, data classification, feature extraction, and comparison with a healthy model. Figure 1 summarizes these successive steps from the raw data to the final graphical feedback.

- The segmentation step uses an adaptive change-point detection algorithm to process IMU recordings. The method searches for significant changes in the time-frequency space at a given scale, i.e., instants where the subject changed their behavior/activity. Signals are thus segmented into several homogeneous regimes that will help to extract knowledge from the global recording.
- Once these homogeneous regimes are segmented, they are classified as walking or non-walking phases through a supervised classification procedure. A second algorithm identifies, within non walking phases, sedentary and non-sedentary regimes, thus providing a full labelization of the regimes. Sedentary regimes correspond to activities that are not walking phases but that imply movements from the recorded subjects (in our case, walking up and down stairs, opening a fire door, and performing a 90° turn). On the other hand, non-sedentary regimes correspond to activities that do not imply movements from participants (in our case, leaning, sitting, standing).
- The next step consists of extracting features from the regimes that have been classified as corresponding to a walking phase. These features were selected in order to assess different aspects of gait (stability, steadiness, sturdiness, and symmetry).
- By using models learned from healthy subjects, each walking regime is then given a score represented by distinct color, allowing visual and intuitive feedback.

### 2.1. Data and Protocols

A total of 21 healthy subjects (33.4 ± 14.42 years old, 10 men and 11 women), 6 patients having undergone or about to undergo an orthopedical surgery (48.75 ± 20.32 years old, 1 man and 5 women), and 3 patients suffering from a neurological pathology due to a cerebral lesion (71.5 ± 8.22 years old, 1 man and 2 women) were measured on a semi-controlled protocol. Informed consent was obtained for all participants.

Subjects were equipped with a Shimmer3 IMU (Shimmer, Dublin, Ireland) on their lower back L5 (via the use of a belt strap) (with sampling frequency $F_{s}$ = 100 Hz, battery life = 39–69 h, storage = 8 GB, ±2 g (to ±16 g) for the accelerometer, ±250 dps (to ±2000 dps) for the gyroscope [10]. Subjects were asked to complete several laps of the Neurophysiology Department at Percy Hospital (a semi-controlled environment) and to perform activities at the end of each lap (climbing up and down some stairs, leaning, sitting, standing) for a total protocol duration of precisely 6 min. Participants can complete different numbers of laps depending on their pathology or walking characteristics. However, subjects must be able to complete at least one full lap in less than 6 min in order to perform at least one end-of-lap sedentary activity. If a registered subject fails to complete a lap of the protocol, he/she is not included in the list of selected subjects. Raw signals are filtered between 0.5 and 5 Hz to remove the noise [20] with a Butterworth bandpass filter (4th order). This experimental protocol was approved by the committee for the protection of individuals (Comité de Protection des Personnes) from the Agence Régionale de Santé (ARS). The ID-RCB number of the committee in which this study is included is 2021-A00087-34. All methods were carried out in accordance with the principles of the Declaration of Helsinki.

**Figure 2:**
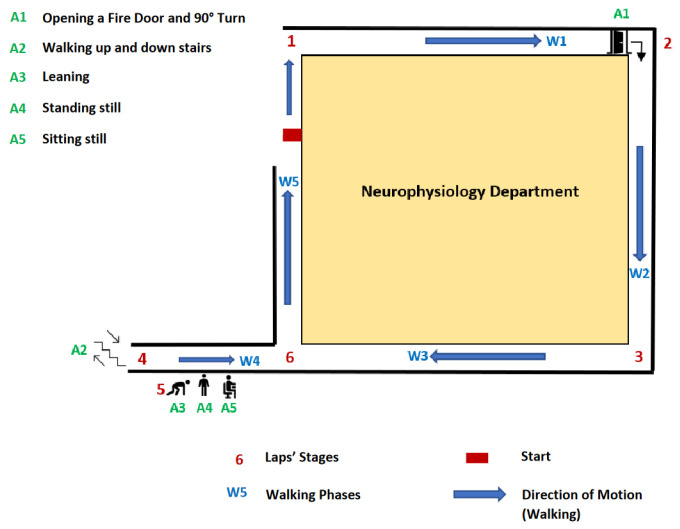
Description of the semi-controlled protocol. Numbers displayed indicate the position of the subject during its path.

The protocol, illustrated on Figure 2, contains several regimes that are either walking phases (denoted **W•**) or activities (denoted **A•**). During each recording, breakpoints were identified thanks to a camera carried by the subject who was left alone to perform the protocol. These breakpoints correspond to transitions between walking phases/activities, activities/walking phases, activities/activities, or walking phases/walking phases. These annotations were conducted collaboratively by two experts and consist of precise timestamps that will be used as ground truth breakpoints’ labels. Characteristics of these transitions are displayed in Table 1.

### 2.2. Step 1: Adaptive Changepoint Detection Method

The first step of the processing pipeline consists of segmenting the raw signals by detecting all activity changes. Intuitively, this segmentation depends on the meaning given to the notion of *change*. In order to adapt the strategy to the signals of interest, we propose to use a supervised approach that learns from annotated data the type of changes that are meaningful in the context and thus the adequate *scale* for the segmentation algorithm.

#### 2.2.1. Data Transformation

In the proposed method, changes are detected in the short-time Fourier transform (STFT) of the raw acceleration/angular velocity signals recorded on the lower back of healthy subjects. This data transformation has been successfully used in multiple publications as it enables visualizing activity changes but also speed changes or event slope changes [21,22,23].

More specifically, two signals of interest are extracted from the raw data (IMU recordings): the craniocaudal angular velocity (gCC) and the anteroposterior acceleration (aAP). These signals were chosen as they are directly influenced by the changes that can be observed during the execution of the protocol (beginning and end of gait, short activities, half turns, and turns). Studies have already used these signals to meet objectives similar to ours (turn detection, detection of activities of daily life) [24,25]. They are normalized (zero mean and unit variance) before being transformed in the time-frequency domain through STFT (3 s window length and 0.1 hop length). Only the 0–5 Hz frequency band, where phenomena of interest are contained, is kept [26,27]. The norms of the STFT coefficients of each aAP and gCC signal are computed and concatenated, providing d=28 frequency bins per frame (14 per signal). The output data will be seen by the segmentation algorithm as a *d*-dimensional multivariate signal.

#### 2.2.2. Changepoint Detection Algorithm

Formally, let $y=\{y_{1},y_{2},\cdots ,y_{n}\}$ denote a $R^{d}$-valued signal with *n* frames: the goal is to detect shifts in the mean of this signal. For *K* change point indexes ${\left\{t_{k}\right\}}_{k=1}^{K}$ ($1<t_{1}<t_{2}<\cdots <t_{K}<n$), a common measure of approximation quality is the empirical quadratic risk:

(1) $$R\left(y,\{t_{k}\}\right):=\sum_{k=0}^{K+1}\left(\sum_{t=t_{k}}^{t_{k+1}-1}{∥y_{t}-{\bar{y}}_{t_{k}\ldots t_{k+1}}∥}^{2}\right)$$

where ${\bar{y}}_{t_{k}\ldots t_{k+1}}$ is the empirical mean of $y_{t_{k}},\ldots ,y_{t_{k+1}-1}$ and $t_{0}:=1$ and $t_{K+1}:=n$ by convention. The risk (1) is simply the error when approximating *y* by a piecewise constant signal. The objective is to find the change points ${\left\{t_{k}\right\}}_{k=1}^{K}$ that minimize this risk. When the number of breaks *K* is not known (which is the case in this article), the empirical quadratic risk is penalized with a linear penalty and the optimal breakpoints can be estimated by solving the following optimization problem:

(2) $$\hat{K},{\hat{t}}_{1},\ldots ,{\hat{t}}_{K}:=\underset{K,t_{1},\cdots ,t_{K}}{\arg\min}\left(\underset{R_{\beta }\left(y,\left\{t_{k}\right\}\right)}{\underset{︸}{R(y,{\left\{t_{k}\right\}}_{k=1}^{K})+\beta K}}\right)$$

where the smoothing parameter β>0 controls the trade-off between model complexity and model accuracy. The value of β is critical: a large β only detects strong breaks (change of activity for instance) whereas a low β also detects small breaks (change within the walking phases). For a fixed β, this discrete optimization problem can be solved efficiently in linear time O(n) with the PELT algorithm [28].

#### 2.2.3. Calibration of β

Instead of manually calibrating this parameter by trial and error, a supervised approach, described in previous studies [29,30], is adopted in this article to learn an optimal value. This method takes as input an annotated database of physiological signals where the relevant breakpoints have been annotated by an expert, and learns the adequate parameter value that is able to reproduce the segmentation strategy of the expert on new signals.

Formally, the input is a collection of *N* labeled signals $y^{\left(1\right)},\ldots ,y^{\left(N\right)}$, and for each $y^{\left(i\right)}$, an expert manually provided the set of true change point indexes ${\left\{t_{k}^{\left(i\right)}\right\}}_{k}$. The optimal smoothing parameter, denoted ${\hat{\beta }}_{\mathrm{opt}}$, is such that the risk of the true expert segmentation on all signals $R_{\beta }\left(y^{\left(i\right)},{\left\{t_{k}^{\left(i\right)}\right\}}_{k}\right)$ is closest to the one of the predicted segmentation on all signals $R_{\beta }\left(y^{\left(i\right)},{\left\{{\hat{t}}_{k}^{\left(i\right)}\right\}}_{k}\right)$:

(3) $${\hat{\beta }}_{\mathrm{opt}}:=\underset{\beta >0}{\arg\min}\frac{1}{N}\sum_{i=1}^{N}\left(R_{\beta }\left(y^{\left(i\right)},{\left\{t_{k}^{\left(i\right)}\right\}}_{k}\right)-R_{\beta }\left(y^{\left(i\right)},{\left\{{\hat{t}}_{k}^{\left(i\right)}\right\}}_{k}\right)\right).$$

Intuitively, the algorithm will search for the penalty β that allows to reproduce the annotations by forcing the β−optimal solution ${\left\{{\hat{t}}_{k}^{\left(i\right)}\right\}}_{k}$ to be as close as possible to the ground truth partition ${\left\{t_{k}^{\left(i\right)}\right\}}_{k}$. The excess penalized risk is a convex function w.r.t. β (precisely, this is an affine function minus a concave function: see [29] for details). We therefore used Brent’s method as a convex optimization tool to minimize this component for each signal.

### 2.3. Step 2: Classification of Segmented Phases

Once the homogeneous regimes have been extracted from the raw data, our aim is to assess whether each of these regimes consists of a walking phase or another activity (sedentary or not). This task is known in the literature as Walking Bout (WB) detection.

There are several existing approaches to perform this detection. The first two approaches are peak detection [31] and step detection methods [32]. These two types of methods impose a significant computational heaviness and are inadequate for FLEs if we wish to provide a simple and easy-to-understand visualization method as we do. Moreover, these types of studies do not allow to characterize all the portions of the signal (only the WBs) as it is desired in our case. A third group of studies integrates the detection of WB into HAR methods via the use of classifiers. To do so, a list of features is extracted on filtered signals in each frame selected by sliding windows. Machine learning classifiers are trained with supervised data (features’ values associated with specifically targeted labels) and then implemented for each portion of the signal. Several classifiers in particular are then used with a very satisfactory accuracy rate: the support vector machine (SVM) [33], random forest [34], decision trees [35] and k-nearest neighbors [36]. This way of detecting WBs using classifiers matches the objectives of our study. Indeed, it will be possible by using this approach to label every portion of the signal and not only the walking regimes (unlike the first two approaches). Moreover, the computational costs are lower than the first two approaches with satisfactory results.

Our classification procedure is therefore based on feature extraction coupled with supervised learning. However, contrary to state-of-the-art methods used for HAR, instead of using (possibly overlapping) frames, we propose to perform this classification at the regime level. The advantages are twofold: first, because of the segmentation procedure, we know that each regime is stationary, which is a valuable theoretical property for computing robust features. Second, the average length of the regimes is often longer than typical frame durations, which provides more data for computing features.

First, we extract for each detected regime an extensive list of both temporal and frequency features such as variances, means, dominant frequencies, and power at dominant frequencies. These features have been chosen and selected in accordance with a recent state-of-the-art article [10] dedicated to activity classification from IMU signals. The features list is presented in Table 2. These 135 features are retained because their computation is convenient for long FLE signals as they do not require any detection of events (heel strikes, toe strikes). PCA is then applied to these 135 features in order to keep 99% of the cumulative explained variance. As for classifiers, we have compared their performance when applied to our signals, and the SVM classifier with linear kernel was retained for our study.

In our pipeline, two classifiers are trained and used in cascade. The first SVM classifier with the linear kernel (Classifier 1) performs a walking/non-walking binary classification, whereas the second SVM classifier with linear kernel (Classifier 2) only considers the non-walking phases detected by Classifier 1 and classifies them as sedentary or non sedentary.

### 2.4. Step 3: Feature Extraction

In our visual summary, a walking regime is assessed according to four standard criteria: stability, steadiness, sturdiness, and symmetry. The four features to be extracted for regime evaluation, detailed in Table 3, are chosen because of their ability to accurately characterize the gait, their recurrent use in the literature, and ease of computation. These features are directly calculated on filtered signals at each regime level before the PCA has been applied.

**Stability** [37,38]: criterion evaluating postural balance and used for instance to prevent falls [39]. Stable walking can be defined as *gait that does not lead to falls despite perturbations* [40]. This aspect is evaluated by using the root mean square ratio computed on the mediolateral acceleration $RMSR_{ML}$. It corresponds to the ratio of the root mean square of the mediolateral accelerations $RMS_{ML}$ to the root mean square vector magnitude computed on all axes $RMS_{A}$ as displayed in (4). RMS evaluates the magnitude of the acceleration on one specific axis. The higher $RMSR_{ML}$ is, the higher the values of mediolateral accelerations tend to be compared to other accelerations. This indicates an instability on the mediolateral axis and therefore a postural instability. Thus, $RMSR_{ML}$ is selected for our study since it has been proven to be uncorrelated with walking speed [38].

(4) $$\begin{array}{cc} RMS_{A} & =\sqrt{RMS_{ML}^{2}+RMS_{CC}^{2}+RMS_{AP}^{2}},\\ RMSR_{ML} & =\frac{RMS_{ML}}{RMS_{A}} \end{array}$$

**Sturdiness** [3,41]: criterion *evaluating gait amplitude* [3]. For instance, sturdiness can be assessed to quantify observed defects in patients with Parkinson’s disease with low amplitude movements [42]. This aspect is evaluated by using the root mean square ratio computed on the anteroposterior acceleration $RMSR_{AP}$ whose computation is conducted the same way it is performed for $RMSR_{ML}$. The higher it is, the higher the anteroposterior accelerations’ values compared to other accelerations and the higher the sturdiness is. Indeed, high anteroposterior acceleration values mean that step impulsions are vigorously performed by the participant. $RMSR_{AP}$ is used instead of $RMS_{AP}$ in order to limit the influence of the walking speed.

(5) $$RMSR_{AP}=\frac{RMS_{AP}}{RMS_{A}}$$

**Figure 3:**
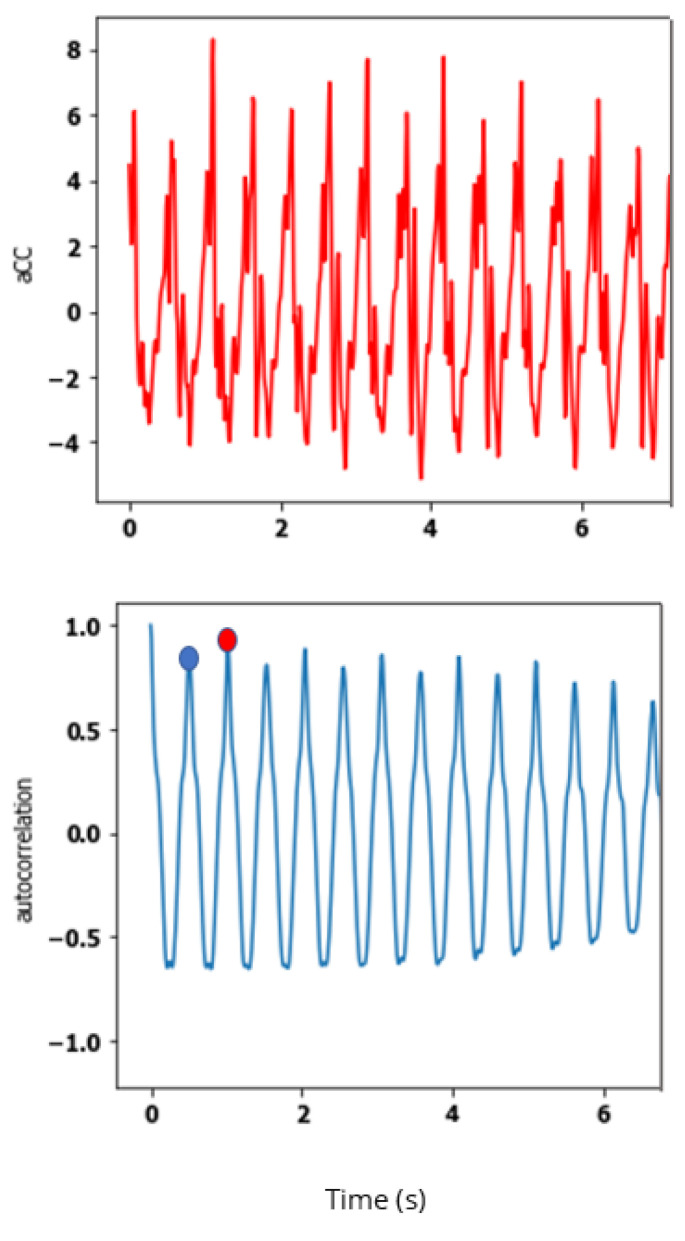
One aCC signal and its associated unbiased autocorrelation. Definition of $P1_{CC}$ (blue dot) and $P2_{CC}$ features (red dot).

**Steadiness** [43,44]: criterion evaluating step regularity, i.e., *similarity of consecutive strides* [45]. This criterion can be used to quantify locomotion flaws in targeted cohorts with lower limb defects (such as transfemoral amputees). This category is evaluated by using the second peak of the autocorrelation coefficients ($P2_{CC}$) calculated on craniocaudal accelerations via the Wiener–Khinchin theorem. This unbiased autocorrelation function uses both fast Fourier transform (FFT) and inverse fast Fourier transform (iFFT) as detailed in Table 3. This feature compares the similarity between strides within a walking regime since it occurs with a time lag of two steps. The higher $P2_{CC}$ is, the more similar the performed strides are. Let aCC be the associated craniocaudal acceleration signal, ConjFFT(aCC) the complex conjugate of FFT(aCC), and ACF the autocorrelation coefficients: $P2_{CC}$ is defined as detailed in (6). Figure 3 shows a craniocaudal acceleration signal associated with its autocorrelation: $P1_{CC}$ and $P2_{CC}$ locations are presented.

(6) ACF=iFFT[FFT(aCC)ConjFFT(aCC)]

**Symmetry** [43,44]: criterion evaluating step symmetry. *A symmetric gait pattern for humans is characterized by the almost identical behavior of bilateral limbs during a gait cycle* [46]. This aspect is evaluated by using the first peak of the autocorrelation coefficients ($P1_{CC}$) calculated on craniocaudal accelerations via the Wiener–Khinchin theorem. It compares the similarity between steps within a walking regime since it occurs with a time lag of one step. $P1_{CC}$ evaluates the ability to maintain vertical correspondence between right and left hemi-bodies during walking regimes. The higher it is, the more similar the steps from both sides are.

### 2.5. Step 4: Score Generation and Graphical Feedback

Using a database of healthy walking phases taken from the healthy subjects from the recorded cohort, statistics for the different features are computed (means, percentiles, etc.). These models are then used to assess each novel walking phase with the scoring procedure described as follows.

Considering a feature with mean μ and standard deviation σ on all walking regimes from healthy subjects, we compute the z-score normalized feature

(7) $$z=\frac{x-\mu }{\sigma }.$$

The z-score normalized features are then displayed with a color bar of boundaries $\left[x_{min},x_{max}\right]$ where $x_{min}$ and $x_{max}$ are, respectively, the 10% and 90% percentiles of the normalized features on the healthy subjects. *Slightly below healthy* values correspond to regimes with z-scores just below those obtained for the 10th percentile of healthy subjects, and *slightly above healthy* values correspond to regimes with z-scores just above those obtained for the 90th percentile of healthy subjects.

### 2.6. Evaluation Metrics

In the next section, we conduct a step-by-step assessment of each step of the pipeline with adapted metrics. All simulations are run with 3-fold cross-validation. To that aim, we split the dataset into three balanced sets (two training sets and one validation set) of seven healthy subjects and three pathological subjects (two participants having undergone or about to undergo orthopedical surgery and one neurological patient). This cross-validation allows one to verify that the algorithm developed in this study can be used on new unseen data to apply the desired segmentation.

#### 2.6.1. Evaluation of the Adaptive Change-Point Detection

The supervised segmentation procedure is assessed with three standard evaluation metrics: precision, recall, and F1-score. A predicted change point is a true positive (TP) if it is close to a true change point (within a specific positive temporal margin). This margin is set to 3.5 s and corresponds to the maximum accepted error for a change point. It must be lesser or equal to the minimum temporal distance between two true change points. Recall which corresponds to the proportion of true change points that are correctly predicted is the ratio of the number of TPs to the number of true change points K∗. Precision is the proportion of predicted change points that are associated with true change points. It is the ratio of the number of TPs to the number of predicted change points *K*.

(8) $$Precision=\frac{TP}{K}\;\;Recall=\frac{TP}{K*}\;\;F1Score=2\frac{PrecisionRecall}{Precision+Recall}$$

#### 2.6.2. Joint Evaluation of Segmentation and Classification Steps

After the first two steps of the pipeline, each data sample is labeled as Walking, Non-Walking/Non-Sedentary, or Sedentary. Intuitively, the sample-scale classification performances depend both on the segmentation step and on the regime classification step. To jointly evaluate these tasks, we compute the confusion matrix between all three labels. Each coefficient of the matrix represents the percentages of samples annotated as belonging to the row activity that have been classified as the column activity. Perfect performances would correspond to a diagonal matrix.

## 3. Results

### 3.1. Adaptive Change-Point Detection

The cross-validation results are the following: **F1-Score** 0.76±0.01, **Recall** 0.79±0.02, and **Precision**0.74±0.02. These are satisfactory results and enable a proper segmentation that can be relied on for the further setup of graphical feedback. The recall and precision results are well-balanced, which means that there is no oversegmentation or undersegmentation.

### 3.2. Joint Evaluation of Segmentation and Classification Steps

**Figure 4:**
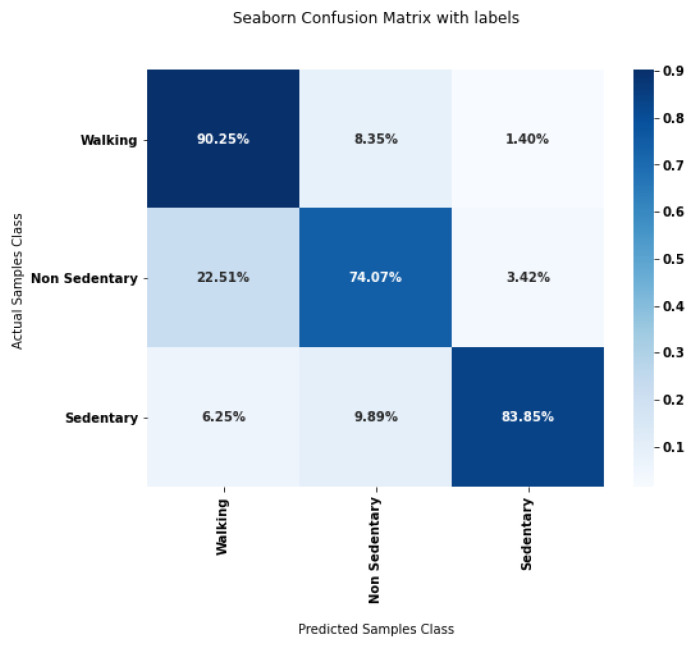
Confusion matrix for the segmentation and classification steps of our processing pipeline.

Figure 4 displays the confusion matrix at sample scale evaluating both segmentation and classification. The correct class (Walking, Non-Sedentary, Sedentary) is accurately predicted for a large majority of samples. In detail, Walking samples are well distinguished from other classes whereas Sedentary and Non-Sedentary samples are less precisely discriminated.

Several HAR methods detailed in the literature attempt to classify WBs. Their results have all been evaluated in a review conducted by the authors of the current article [10]. Table 4 displays accuracy values for different kinds of classifiers used in recent HAR studies carried out in free-living conditions as well for these same classifiers tested on our retrieved data. Accuracy results are satisfactory for our implemented chosen linear SVM cascade classifiers (0.88) when compared to these results (no other accuracy result above 0.89). It is noteworthy that the majority of these methods are difficult to compare with ours because of variations in the conditions of application of the classifiers (very few studies compute the training features at the scale of a regime for instance, sensors are placed on different locations).

### 3.3. Scores and Graphical Feedback

**Figure 5:**
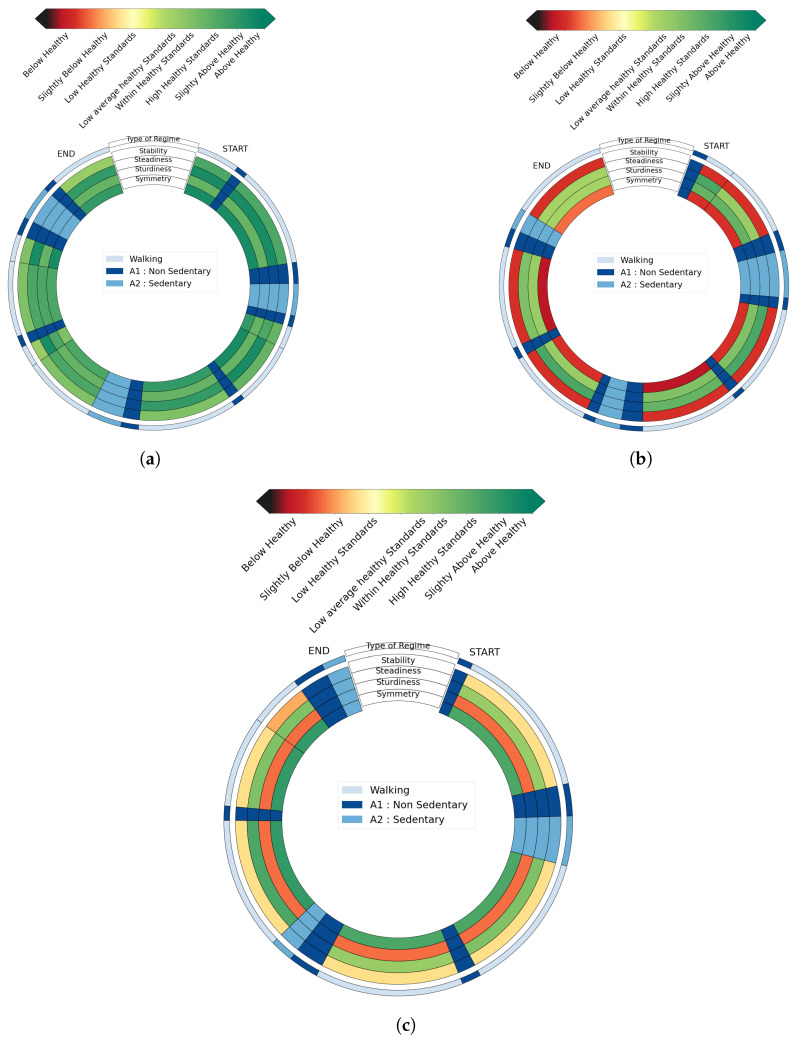
Graphical feedbacks from **(a)** *HSU*, **(b)** *PSU1*, **(c)** *PSU2*.

**Figure 6:**
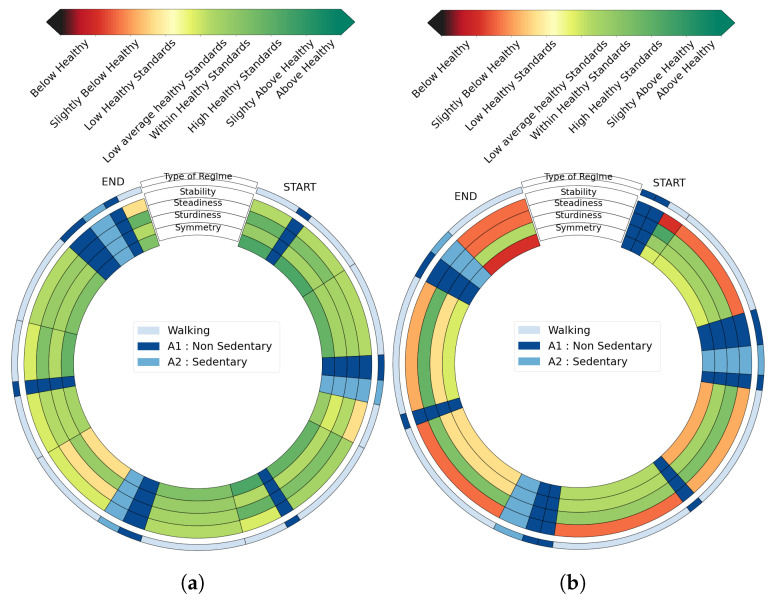
Evaluation of pre-hospitalization acquisition and post-hospitalization acquisition for a subject who has undergone knee ligamentoplasty. Post-operation evaluation displays a worse state. (**a**) Pathological Subject 3 graphical feedback **Pre-surgery** *PSU3A*; (**b**) Pathological Subject 3 graphical feedback: **Post-surgery** *PSU3B*.

Graphical outputs are plotted for four subjects: one 23 year-old healthy male subject (*HSU*—Figure 5a), one 66 year-old female pathological subject (*PSU1*—Figure 5b) with a chronic gluteus medius insufficiency, one 75 year-old woman with post-radiation left brachial plexitis called *PSU2*, and one 25 year-old female pathological subject (*PSU3*—Figure 6b) in an immediate post-operative phase of a knee ligamentoplasty. For the *PSU3* subject, we have also computed the visual feedback in the immediate pre-operative phase of her knee ligamentoplasty as displayed in Figure 6.

These graphs display the evaluation of the whole protocol segmented in regimes in a clockwise manner. The first outer circle specifies the nature of the segmented regimes: dark blue for non-sedentary activities, standard blue for sedentary activities, and light blue for walking regimes. The four next inner concentric circles are each associated with a gait criterion: stability, steadiness, sturdiness, and symmetry. Each portion of these circles delimited by black lines corresponds to a segmented regime, whose length is proportional to the duration of the regime. For a given evaluation criterion, each walking regime is then assigned a color from dark red to dark green. This color depends on the comparison of this regime to the average healthy walking regime. Non-walking regimes are not evaluated and are displayed in dark blue for non-sedentary activities and light blue for sedentary activities (as in the first outer circle).

Several interesting features can be highlighted by simple visual inspections of these diagrams. We see for instance in the feedback obtained for *HSU* (Figure 5a) that walking regimes from this participant are deemed to be of satisfactory quality according to all evaluated criteria. On the other hand, scores’ figures for several walking regimes from *PSU1* and *PSU2* are below average healthy standards. For PSU1, stability and symmetry (first and fourth inner circles) are degraded whereas *PSU2* displays deteriorated stability and sturdiness (first and third inner circles). The tool thus allows for a simple interindividual comparison, which is both quantitative (thanks to the colors) and qualitative (thanks to the four criteria). Furthermore, the temporality of the whole excercice is preserved, allowing for a better understanding and interpretation. Figure 6 shows another potential use for the tool in the context of longitudinal follow-up. By comparing the diagram obtained before (Figure 6a) and after surgery (Figure 6b) from participant *PSU3*, it is visible that the post-surgery consequences mostly affect the stability of the locomotion, and that symmetry is also degraded.

## 4. Discussion

### 4.1. Performances

The metrics obtained for the supervised segmentation are satisfactory (F1-score around 75%) and the observed margin of error is due either to annotation approximations or specific breakpoints difficult to detect because of the movement of the subjects (quick transitions or turns that are taken so quickly that they do not appear as a clear breakpoint). A study differentiating the results obtained according to the types of transitions was carried out. Turns appear to be less accurately detected than transitions between slow/steady regimes and active regimes (A3/A4/A5 to W4 for example, see Table 1 for the categories of changes). This is due to the fact that these turns are not performed the same way by all subjects. Some are too fast to produce a particular pattern in the spectrogram used by our method. This may explain the few errors observed in the segmentation. The segmentation is currently performed using spectrograms whose hop size is 0.1 s, thus limiting the temporal resolution that can be achieved. In case we would like to lower it, it is possible to do so, but at the expense of the computation time.

As for the joint segmentation/classification assessment, the walking phases are well-predicted (>90%) and sufficiently discriminated for the two other phases (>75% for non-sedentary phases and >83% for sedentary phases). Again, imprecision margins are consequences of errors in the segmentation. In addition to segmentation error, inaccuracy can be introduced by the two classifiers. Walking regimes are well detected because this activity is structured and made up of repetitive and precise patterns that therefore manifest themselves with intense spectral signatures. Since a significant proportion of the features used for classification are spectral features, this probably facilitates the classification process. Sedentary and non-sedentary activities are inherently more difficult to differentiate. For example, activities where subjects open fire doors (A1) include movements similar to those observed during walking (stomping and some slow steps) that have spectral signatures closer to those of walking activities. As a result, non-sedentary activities are often mistaken for walking regimes. On the contrary, the intensity of sedentary activities tends to be very low, which may lead to confusion with some low-energy non-sedentary activities. All of these confusions are often encountered in other studies aimed at classifying activities and especially walking activities [52].

### 4.2. Robustness of the Features

**Figure 7:**
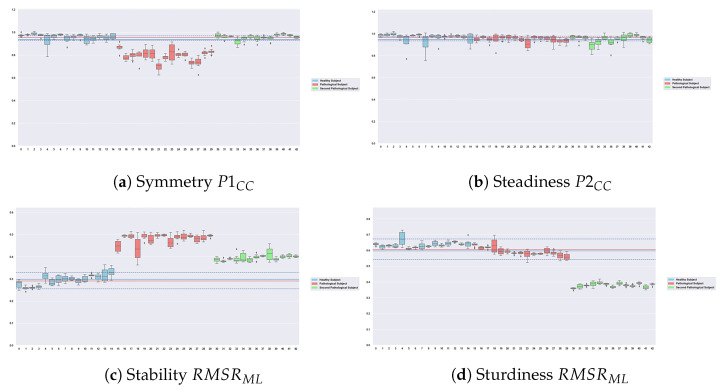
Evaluation of the robustness of selected features. Features with low dispersion and high discrimination between classes. The blue horizontal line shows the average value of the feature for all healthy subjects, the red horizontal line shows the median value of the feature for all healthy subjects, and the dotted lines correspond to the 75th/25th percentiles. Each boxplot corresponds to 10 computations of the feature on a walking regime on 10 degraded ranges. Boxplots are displayed with specific colors depending on their associated subject: blue for a healthy subject, red for *PSU1*, and green for *PSU2*.

In the final graphical feedback, four features are used to characterize the gait activity. The robustness of the feedback depends mainly on the robustness of those features, especially when confronted with segmentation errors. To investigate this issue, we conducted an additional experiment where we intentionally degrade the segmentation process (e.g., by voluntarily lowering the number of samples for feature computation), in order to assess the robustness of the features. In total, 10 degraded configurations are tested, as described in Table 5. Figure 7 shows the distribution of features in all categories over the 10 configurations in walking sections of one healthy subject (*HSU*) and two pathological subjects: *PSU1* (gluteus medius deficiency) and *PSU2* (post-radiation left brachial plexitis). *PSU1* has shown the highest instability and lack of symmetry in their deambulation, *PSU2* has shown degraded sturdiness. For each subject, we have extracted all walking regimes, and computed the features according to the different configurations. Each box contains the distribution of the different values of this feature on all 10 tested configurations in a given walking regime. The walking regimes for *HSU* are displayed in blue, and the ones of the first, second, and third pathological subject, respectively, in red and green. The blue horizontal line shows the average value of the feature computed on all 10 degraded ranges of every walking regime from all healthy participants (dotted lines correspond to the 75th/25th percentiles). Thus, the distribution for one healthy subject and two pathological subjects as well as the average distribution for all healthy participants are shown in Figure 7.

One first observation is that all boxes display little spread over all recorded subjects, which suggests that the computation process is robust. It is interesting to note that the differences between the three subjects are clearly visible for all walking regimes in all categories except for steadiness (no recorded participants displayed an affected regularity). Moreover, the patient with the most impact on their stability (*PSU1*) displays boxes associated with the $RMSR_{ML}$ feature that are even more detached than *PSU2* from the figures of the healthy subjects, which confirms the different visual impacts observed on the gait of each pathological subject. This feature thus presents satisfactory robustness results in terms of dispersion on the degradation ranges as well as in terms of discrimination between subjects. This confirms the relevance of using this feature to evaluate the stability of walking regimes. The calculation of this feature remains indeed constant on all the ranges presented in Table 5, which allows our method to be correctly applied despite eventual segmentation errors that may occur. No patient presented a continuous affection in steadiness and it was thus difficult to estimate the discrimination power from $P2_{CC}$: it must be evaluated in further works. Other figures and additional experiments show that all other features listed in Table 3 display the same consistency and robustness, which is an important asset of our proposed approach.

### 4.3. Relevance of the Graphical Feedback and Possible Usecases

Several important conclusions can be made from the graphical feedbacks obtained on the *HSU*, *PSU1*, *PSU2*, and *PSU3* subjects.

***The overall readings of the graphs from HSU in Figure 5a, PSU1 in Figure 5b, and PSU2 in Figure 5c correlate with field observations of subjects’ deambulations made by an orthopedic surgeon and a neurologist (Hôpital d’Instruction des Armées Percy)***: *PSU1* suffers from gluteus medius insufficiency causing Trendelenburg-type lameness because of multiple right hip surgeries. This lameness is related to the low scores obtained for symmetry and stability for this participant. As for *PSU2*, this patient suffered from post-radiation left brachial plexitis 20 years after radiation treatment for breast cancer. Complete paralysis of the entire left upper limb was observed. The patient’s stability and sturdiness are accordingly affected. The displayed impairment of stability may be due to the imbalance related to the dead weight of her left arm, which hangs from the shoulder and weighs at least 10 kg. Besides, *PSU2* performed steps with little amplitude which explains degraded scores in sturdiness as displayed in the visual feedback (third inner circle). This confirms that this graphical tool gives a correct overall perception of the subjects’ gait actual defects (this must nevertheless be extended to other subjects).

***Longitudinal follow-ups allowed by these visual feedbacks accurately reflect observed degradations or improvements in subjects’ walking regimes:*** the differences of the same subject before and after their knee ligamentoplasty surgery are presented in Figure 6. In this figure, an obvious degradation is observable between the two recordings as detailed in Section 2.4. This corresponds to the visual observation made by the surgeon: the subject presented a much more degraded gait after surgery than before. After the surgery, there was a significant quadricipital sideration which fully explains the alteration in stability and symmetry (first and fourth inner circles in graphical outputs). This shows how efficient this graphical tool is to track a patient’s physical activity longitudinally.

***Visual feedbacks provide time scales of segmented regimes and allow for a new type of ambulatory gait analysis***: regimes’ delineations implemented with black lines described in Section 2.5 allow the relative lengths of each segmented regimes to be assessed and compared with each other. This provides a new prism for innovative macro analysis when associated with the evaluations that these visual feedbacks offer: long sedentary regimes might induce better stability scores for walking regimes starting afterward since it can remove fatigue symptoms, for instance.

To the authors’ knowledge, few studies based on the use of IMUs in FLEs have endeavored to provide a macro analysis displayed in the form of an easy-to-understand visual legend that fully assesses the entire timeline of FLE signals. Provided summaries either focus on metrics that are too specific, which prevents clear and didactive visual feedback, or on metrics that are too general, which prevent a complete assessment of a subject’s physical activity in free environments. In these kinds of studies, the influence of time is often erased by computing features that are often agglomerated over the whole of the measurements as explained above. Rather than knowing the percentage of time spent on each activity, we can, for example, be interested in the impact of transitions between activities on the quality of walking regimes, on the evolution of this quality over several consecutive regimes. In this section, we developed a graphical tool by circumventing these pitfalls of physical activity assessment: time-scales of segmented regimes are provided as well as a continuous evaluation of the physical activity over several walking regimes thanks to pre-defined criteria. Our graphical tool will enable refined follow-ups, displaying an enhanced macro analysis of gait phases. The output graphs allow for easier and more meaningful intra- and interindividual comparisons than those allowed by the global monitoring metrics generally used in the literature. This graphical tool could also allow practitioners to quickly determine the areas of instability in their patients, to identify the influences of fatigue, and perform longitudinal follow-ups allowing new interpretations. Besides, it could help to evaluate rehabilitation procedures or treatment choices for specific diseases and to assess the impact of treatments on pathologies such as musculoskeletal tumors of the lower limbs or neurological disorders (Parkinson’s disease, for instance)

## 5. Conclusions

A pipeline aiming to provide practitioners with a graphical evaluation of their subjects walking in semi-FLEs is presented here. These methods are innovative in the interpretations they offer, adapting a notably concerning segmentation which follows the annotation strategies, precise and ergonomic in its final visual rendering. The rendered visual summary will help practitioners to provide a reliable comprehensive longitudinal tracking of locomotion in free environments. This could for instance allow one to improve post-operatory follow-ups and evaluate rehabilitation procedures or treatment choices for specific diseases. The first results are encouraging since they correlate closely with field observations of the walking state of the recorded subjects. Besides, additional criteria such as time scales can enable enhanced interpretations (evaluation of fatigue’s impact, development of transitions between activities). However, these methods need to be tested on longer signals in order to define whether they can be applied with the same efficiency to signals collected in FLEs.

## Figures and Tables

**Table 1:** Details of annotated transitions.

Transition Identification	Details	Type of Regime
W1	Walk (1 → 2)	Walking
A1	Door opening and 90-degree turn	Non-Sedentary
W2	Walk (2 → 3)	Walking
W3	Walk (3 → 4)	Walking
A2	Going up 3 stairs U-turn and going down 3 steps stairs	Non-Sedentary
A3	Leaning	Sedentary
A4	Standing Still	Sedentary
A5	Sitting Still	Sedentary
W4	Walk (5 → 6)	Walking
W5	Walk (6 → 1)	Walking

**Table 2:** Details of features used for classification. For each formula, $X=[x_{1},x_{2}\ldots ,x_{n}]$ is assumed to be one of the six dimensions (three linear accelerations and three angular velocities) from the IMU signal. In total, 135 features are used. Notations: $\bar{x}$ is the empirical mean of *X*, $\hat{\sigma }$ is the empirical unbiased standard deviation of *X*, FFT(*X*) is the Fourier transform of X, ConjFFT(*X*) denotes the complex conjugate of FFT(*X*).

Features	Signal Used for Computation	Description	Domain	Formulas
mean_signal	All 6 Signals	Mean	Time	$\bar{x}$
std_signal	All 6 Signals	Standard deviation	Time	$\hat{\sigma }$
var_signal	All 6 Signals	Variance	Time	${\hat{\sigma }}^{2}$
min_signal	All 6 Signals	Minimum	Time	min(x)
max_signal	All 6 Signals	Maximum	Time	max(x)
PD0_signal	All 6 Signals	Power at the first dominant frequency	Frequency	$max(\frac{FFT(X)*ConjFFT(X)}{N})$
F0_signal	All 6 Signals	First dominant frequency	Frequency	$argmax(\frac{FFT(X)*ConjFFT(X)}{N})$
PD2_signal	All 6 Signals	Power at the second dominant frequency	Frequency	$second\_max(\frac{FFT(X)*ConjFFT(X)}{N})$
F2_signal	All 6 Signals	Second dominant frequency	Frequency	$second\_argmax(\frac{FFT(X)*ConjFFT(X)}{N})$
CV_signal	All 6 Signals	Coefficient of variation	Time	$\hat{\sigma }/\bar{x}$
p75_signal	All 6 Signals	75th percentile	Time	Let R be the 75th percentile rank: $R=\frac{75*n}{100}$, p75 corresponds to the Rth value on the sorted X array
p25_signal	All 6 Signals	25th percentile	Time	Let R be the 25th percentile rank: $R=\frac{25*n}{100}$, p25 corresponds to the Rth value on the sorted X array
p85_signal	All 6 Signals	85th percentile	Time	Let R be the 85th percentile rank: $R=\frac{85*n}{100}$, p85 corresponds to the Rth value on the sorted X array
p15_signal	All 6 Signals	15th percentile	Time	Let R be the 15th percentile rank: $R=\frac{15*n}{100}$, p15 corresponds to the Rth value on the sorted X array
p95_signal	All 6 Signals	95th percentile	Time	Let R be the 95th percentile rank: $R=\frac{95*n}{100}$, p95 corresponds to the Rth value on the sorted X array
p5_signal	All 6 Signals	5th percentile	Time	Let R be the 5th percentile rank: $R=\frac{5*n}{100}$, p5 corresponds to the Rth value on the sorted X array
p75m_signal	All 6 Signals	75th percentile at the middle of the signal (2/3 of the signal)	Time	Let R be the 75th percentile rank: $R=\frac{75*n}{100}$, p75 corresponds to the Rth value on the sorted X array
p25m_signal	All 6 Signals	25th percentile at the middle of the signal	Time	Let R be the 25th percentile rank: $R=\frac{25*n}{100}$, p25 corresponds to the Rth value on the sorted X array
p85m_signal	All 6 Signals	85th percentile at the middle of the signal	Time	Let R be the 85th percentile rank: $R=\frac{85*n}{100}$, p85 corresponds to the Rth value on the sorted X array
p15m_signal	All 6 Signals	15th percentile at the middle of the signal	Time	Let R be the 15th percentile rank: $R=\frac{15*n}{100}$, p15 corresponds to the Rth value on the sorted X array
RMS_signal	All 6 Signals	Root mean Ssquare	Time	$RMS=\sqrt[{}]{\frac{1}{n}*\left(\sum_{i=1}^{n}x_{i}^{2}\right)}$
P1_aCC	aCC	First peak of autocorrelation coefficients for craniocaudal acceleration	Time	ACF=iFFT[FFT(X)∗ConjFFT(X)], P1 is the first peak of ACF
P2_aCC	aCC	Second peak of autocorrelation coefficients for craniocaudal acceleration	Time	ACF=iFFT[FFT(X)∗ConjFFT(X)], P2 is the second peak of ACF
VM	All 6 Signals	Vector magnitude of all accelerations (craniocaudal aCC, mediolateral aML, and anteroposterior aAP)	Time	$VM=\sqrt{aCC^{2}+aAP^{2}+aML^{2}}$

**Table 3:** Features used to establish scores for the graphical feedback. In total, four features are used. ConjFFT(*X*) denotes the complex conjugate of FFT(*X*).

Categories	Features	Description	Mathematical Computation
Steadiness	$P2_{CC}$	The second peak of the autocorrelation coefficients calculated on craniocaudal accelerations via the Wiener–Khinchin theorem: the higher it is, the more similar the steps are.	ACF=iFFT[FFT(X)ConjFFT(X)], P1 is the first peak of ACF whereas P2 is the second peak
Symmetry	$P1_{CC}$	The first peak of the autocorrelation coefficients calculated on craniocaudal accelerations via the Wiener–Khinchin theorem: the higher it is, the more similar the strides are.	P1 is the first peak of ACF whereas P2 is the second peak
Sturdiness	$RMSR_{AP}$	Root mean square ratio on anteroposterior acceleration. The higher it is, the higher the sturdiness is.	$RMS_{A}=\sqrt{RMS_{ML}^{2}+RMS_{CC}^{2}+RMS_{AP}^{2}}$, $RMSR_{AP}=\frac{RMS_{AP}}{RMS_{A}}$
Stability	$RMSR_{ML}$	Root mean square ratio on mediolateral acceleration. The lower it is, the higher the stability is.	$RMS_{A}=\sqrt{RMS_{ML}^{2}+RMS_{CC}^{2}+RMS_{AP}^{2}}$, $RMSR_{ML}=\frac{RMS_{ML}}{RMS_{A}}$

**Table 4:** Average accuracy values for different kinds of classifiers used in the literature to classify activities (including walking bouts) in free environments.

Type of Classifiers	Reported Performances	Performances on Our Data
Support Vector Machine SVM	0.72 [47]	0.88 ± 0.14
	0.85 [14]	
	0.74 [48]	
Random Forest	0.88 [49]	0.85 ± 0.16
	0.88 [50]	
	0.86 [14]	
Decision Tree	0.82 [47]	0.77 ± 0.17
	0.83 [51]	
	0.80 [14]	
k Nearest Neighbors	0.75 [47]	0.89 ± 0.06
	0.74 [49]	
	0.68 [50]	

**Table 5:** Degraded configurations for the computation of the features.

Configurations
All the regime is used (normal configuration)
Only the first 3 s of the regime are used
Only the first 3.5 s of the regime are used
Only the first 4 s of the regime are used
Only the first 5 s of the regime are used
Only the first 40% of the regime is used
Only 40% of the regime is used (with start at 20% of the total duration)
Only 40% of the regime is used (with start at 30% of the total duration)
Only 40% of the regime is used (with start at 40% of the total duration)
Only the last 40% of the regime is used

## Data Availability

The data generated during this study are not publicly available due to ethical restriction regarding subjects’ personal information but are partially available from the corresponding author upon reasonable request.

## Abbreviations

The following abbreviations are used in this manuscript:

FLE	Free-Living Environment
Semi FLE	Semi Free-Living Environment
HAR	Human Activity Recognition
IMU	Inertial Measurement Unit
